# Resistin in idiopathic inflammatory myopathies

**DOI:** 10.1186/ar3836

**Published:** 2012-05-11

**Authors:** Mária Filková, Hana Hulejová, Klára Kuncová, Lenka Pleštilová, Lucie Andrés Cerezo, Heřman Mann, Martin Klein, Josef Zámečník, Steffen Gay, Jiří Vencovský, Ladislav Šenolt

**Affiliations:** 1 Institute of Rheumatology, Department of Experimental Rheumatology of the 1st Faculty of Medicine, Charles University in Prague, Na Slupi 4, Prague 2, 128 50, Czech Republic; 2 Department of Pathology and Molecular Medicine, 2nd Faculty of Medicine, Charles University in Prague, V Úvalu 84, Prague 5, 150 06, Czech Republic; 3 Center of Experimental Rheumatology, University Hospital Zurich, Gloriastrasse 23, Zurich, CH-8091, Switzerland

## Abstract

**Introduction:**

The purpose of this study was to evaluate and compare the serum levels and local expression of resistin in patients with idiopathic inflammatory myopathies to controls, and to determine the relationship between resistin levels, inflammation and disease activity.

**Methods:**

Serum resistin levels were determined in 42 patients with inflammatory myopathies and 27 healthy controls. The association among resistin levels, inflammation, global disease activity and muscle strength was examined. The expression of resistin in muscle tissues from patients with inflammatory myopathies and healthy controls was evaluated. Gene expression and protein release from resistin-stimulated muscle and mononuclear cells were assessed.

**Results:**

In patients with inflammatory myopathies, the serum levels of resistin were significantly higher than those observed in controls (8.53 ± 6.84 vs. 4.54 ± 1.08 ng/ml, *P *< 0.0001) and correlated with C-reactive protein (CRP) levels (r = 0.328, *P *= 0.044) and myositis disease activity assessment visual analogue scales (MYOACT) (r = 0.382, *P *= 0.026). Stronger association was observed between the levels of serum resistin and CRP levels (r = 0.717, *P *= 0.037) as well as MYOACT (r = 0.798, *P *= 0.007), and there was a trend towards correlation between serum resistin and myoglobin levels (r = 0.650, *P *= 0.067) in anti-Jo-1 positive patients. Furthermore, in patients with dermatomyositis, serum resistin levels significantly correlated with MYOACT (r = 0.667, *P *= 0.001), creatine kinase (r = 0.739, *P *= 0.001) and myoglobin levels (r = 0.791, *P *= 0.0003) and showed a trend towards correlation with CRP levels (r = 0.447, *P *= 0.067). Resistin expression in muscle tissue was significantly higher in patients with inflammatory myopathies compared to controls, and resistin induced the expression of interleukins (IL)-1β and IL-6 and monocyte chemoattractant protein (MCP)-1 in mononuclear cells but not in myocytes.

**Conclusions:**

The results of this study indicate that higher levels of serum resistin are associated with inflammation, higher global disease activity index and muscle injury in patients with myositis-specific anti-Jo-1 antibody and patients with dermatomyositis. Furthermore, up-regulation of resistin in muscle tissue and resistin-induced synthesis of pro-inflammatory cytokines in mononuclear cells suggest a potential role for resistin in the pathogenesis of inflammatory myopathies.

## Introduction

The inflammatory myopathies are a group of acquired skeletal muscle diseases that include polymyositis (PM), dermatomyositis (DM), inclusion body myositis, and overlap and cancer-associated myositis. Inflammatory myopathies are clinically characterised by proximal muscle weakness and skin changes in DM. An autoimmune origin of inflammatory myopathies is supported by their association with other autoimmune diseases, the presence of autoantibodies, the involvement of histocompatibility genes, the evidence of T-cell-mediated myotoxicity or complement-mediated microangiopathy, and their responses to immunotherapy [1]. Both diseases are also characterised by mononuclear inflammatory cell infiltration in skeletal muscle tissue, muscle fibre necrosis and regeneration. The inflammatory infiltrates in the muscle tissue of DM patients primarily include CD4+ T cells, B cells and dendritic cells in predominantly perimysial distribution, while affected muscle tissue in PM patients is characterised by endomysial presence of CD8+ T cells and macrophages, which often surround and invade non-necrotic muscle fibres [1]. The pathogenesis of inflammatory myopathies has not yet been completely elucidated, but several cytokines and chemokines produced by immune cells and myocytes have already been shown to be involved in the process of muscle tissue damage during myositis [reviewed in [2]].

Resistin, also known as adipocyte-secreted factor (ADSF) or found in inflammatory zone 3 (FIZZ3), is a member of the adipokine family. Originally, resistin was found in adipocytes to induce insulin resistance in mice. It has been associated with several metabolic disorders but also with cancer, inflammatory and immune-mediated diseases [3]. Resistin is up-regulated by inflammatory mediators in peripheral blood mononuclear cells (PBMC) and induces the expression of pro-inflammatory cytokines, such as interleukin (IL)-6, IL-8, monocyte chemoattractant protein (MCP)-1 and tumour necrosis factor (TNF)-α, angiogenic factors and extracellular matrix metalloproteinases, suggesting a broad contribution to many pathological conditions [4-11].

Therefore, we assessed the serum resistin level and its expression in muscle tissues from patients with idiopathic inflammatory myopathies and studied the potential role of resistin in the pathogenesis of muscle tissue damage.

## Materials and methods

### Patients characteristics

Our study consisted of 42 patients with inflammatory myopathies and 27 healthy controls. All patients underwent muscle biopsy that was guided by positive magnetic resonance imaging (MRI) findings from affected muscles [12]. The specific pattern of muscle biopsy, including the immunohistological [13,14] and clinical investigation, showed that 17 patients suffered from DM and 25 from other types of myositis. Based on the novel clinicoserological criteria [15], four patients with myositis could be classified as pure PM, while 21 had overlap myositis. These patients had at least one clinical overlap feature and/or an overlap antibody [15].

Patients were recruited from the inpatient and outpatient departments of the Institute of Rheumatology in Prague. Disease activity was assessed using myositis disease activity assessment visual analogue scales (MYOACT) that globally score constitutional, articular, cardiac, pulmonary, gastrointestinal, cutaneous, muscle organs or systems [16]. Manual muscle testing of eight muscle groups (MMT8) was performed and included one axial, five proximal (two upper extremity, three lower extremity), and two distal muscles (one upper, one lower extremity) [17]. Written informed consent from each participant was obtained prior to enrolment, and the study was approved by the local ethics committee.

### Laboratory measurements

Peripheral blood was obtained from all patients at the time of clinical assessment and from healthy donors. A routine laboratory analysis was performed on fresh serum of patients with inflammatory myopathies. C-reactive protein (CRP) levels in patients and healthy controls were determined by an immuno-turbidimetric technique using an Olympus biochemical analyser (model AU 400, Tokyo, Japan). Creatine kinase and myoglobin levels were measured by routine laboratory methodology using Olympus analyser. All collected serum was stored at -80°C until further analysis. Serum resistin levels were measured with commercially available ELISA according to the manufacturer's protocol (Biovendor, Brno, Czech Republic). The levels of IL-1β, IL-6, TNFα and MCP-1 (RayBiotech, Norcross, GA, USA), along with the levels of perforin (Abcam, Cambridge, UK) and granzyme (Abcam) in cell culture supernatants were measured using commercially available ELISA kits. The absorbance was measured at 450 nm using an ELISA reader (Tecan Sunrise, Salzburg, Austria).

### Immunohistochemistry

Samples for immunohistochemistry were obtained from patients with DM (*n *= 5) and myositis (*n *= 5) at the time of diagnostic muscle biopsy. In these patients, the muscle biopsy was guided by positive magnetic resonance imaging (MRI) findings from affected muscle as previously described [12]. Muscle tissue samples from patients with myasthenia gravis (MG, *n *= 5) were used as non-inflammatory controls. Muscle tissues were snap frozen in isopentane (2-ethylbutane, Sigma-Aldrich, St. Louis, MO, USA) cooled in liquid nitrogen. For the purposes of immunohistochemistry, 5-μm frozen sections were fixed in acetone and 4% paraformaldehyde and blocked with 0.3% H_2_O_2_. The sections were incubated in tris buffered saline (TBS) buffer and incubated with a monoclonal anti-resistin antibody (Phoenix Pharmaceuticals, Inc., Burlingame, CA, USA) at a dilution 1:500 for one hour. Afterwards, the slides were rinsed again in TBS buffer. Antigen-antibody complexes were visualised with a Histofine detection system (Nichirei Biosciences Inc., Tokyo, Japan) using 3, 3'diaminobensidine as a chromogene. Rabbit IgG (Dako Cytomation, Glostrup, Denmark), diluted 1:1,000, was used as a negative control. The sections were slightly counterstained with Harris' hematoxylin. All sections were analysed semiquantitatively by two experienced pathologists who were blinded to the clinical data. The intensity of resistin expression was scored on a four-point scale (0 to 3). A staining intensity of 0 represented a negative result, and scores of 1 to 3 represented weak, moderate and strong positive staining intensity, respectively.

### Cell culture and stimulation assays

Human skeletal muscle cells (Lonza, Basel, Switzerland) were cultured at a density of 100,000 cells in a six-well plate in 2 ml DMEM (Gibco, Carlsbad, CA, USA). PBMC were isolated by standard Ficoll density gradient centrifugation from blood samples donated by healthy donors. Freshly isolated PBMC were resuspended at a density of 10^6 ^cells per well in a 12-well plate in 2 ml of RPMI 1640 (Gibco, Carlsbad, CA, USA). Both myocytes and PBMC were stimulated with human recombinant resistin (Biovendor, Modrice, Czech Republic) at a concentration of 10, 100 or 1,000 ng/ml for 6 and 48 hours at 37°C in 5% CO_2 _humidified atmosphere. In the initial experiment, PBMC were stimulated with either resistin (1,000 ng/μl) alone or in combination with polymyxin B sulphate (5 ug/ml, Sigma-Aldrich, St. Louis, MO, USA) to exclude possible endotoxin contamination. For the gene expression analysis, cells were lysed in RLT buffer (Qiagen, Hilden, Germany) 6 h after stimulation. The supernatants were collected following 48 h of stimulation. The samples were stored at -80°C until use.

### RNA isolation and TaqMan Real-Time PCR

Total RNA was isolated using a MagNA Pure Compact RNA Isolation Kit for the MagNA Pure Compact Instrument (Roche Diagnostics, Mannheim, Germany), and reverse transcription was performed with a High Capacity cDNA Reverse Transcription Kit (Applied Biosystems, Foster City, CA, USA). Real-time PCR was performed using gene expression assays (Applied Biosystems), and the reaction was performed using a 7900HT Fast Real-Time PCR System (Applied Biosystems). Data were analysed using the ddCt method for relative quantification, and 18S was used as an endogenous control.

### MTT assay

The evaluation of the proliferation of myocytes after 72 h stimulation with resistin (10, 100, 1,000 ng/ml) was carried out with an MTT test using dimethylthiazol diphenyl tetrazolium bromide (Sigma-Aldrich). The absorbance was measured at 570 nm via ELISA reader (Tecan Sunrise, Salzburg, Austria).

### Statistical analysis

Data are expressed as the mean ± SD or mean ± SEM where indicated. The Mann-Whitney U test was used for comparisons between two variables, and the Kruskal-Wallis test, along with Dunn's multiple comparison tests, was used for comparisons among more than two variables. Paired T-tests were used for statistical analyses of gene expression. Spearman's and Pearson correlation coefficients were used to correlate any two variables with non-normal and normal distribution, respectively. *P-*values less than 0.05 were considered statistically significant. The analysis and the graphs were performed using GraphPad Prism 5 (version 5.02; GraphPad Software, La Jolla, CA, USA).

## Results

### Characteristics of patients

The characteristics of patients and healthy controls included in this study are given in Table 1. Our study group included 17 muscle biopsy-verified patients with DM and 25 with other types of myositis. Out of these, four had pure PM while 21 fulfilled criteria for overlap myositis. The mean duration of myopathy symptoms ranged from 1 to 154 months. Proximal muscle weakness occurred in 95% of patients. Rash was present in 40%, heliotrope rash in 30%, Gottron's papules/signs in 28%, V sign rash in 28%, shawl sign in 14% and mechanics hands in 35% of patients. The Raynaud phenomenon was present in 21% of patients, arthritis in 26% and dysphagia in 49%. Myositis-associated interstitial lung disease and cardiac involvement was diagnosed in 44% and 23% of patients with inflammatory myopathies. Eight patients were assessed prior to treatment initiation, and 23 patients were treated for less than one month. All other patients underwent treatment for more than one month. In the 29 patients treated with glucocorticoids, the median dose was 40 mg of prednisone or its equivalent/day ranging from 7.5 to 85 mg/day. Seven patients received methotrexate, one patient received methotrexate in combination with cyclosporine A and one patient was treated with azathioprine.

**Table 1:** Characteristics of patients with idiopathic inflammatory myopathies and healthy controls

Characteristic	IIM	HC
N	42	27
Age (years)	53.8 ± 14.2	49.5 ± 8.6
Sex (F/M)	31/11	22/5
CRP (mg/l)	9.3 ± 21.6	1.8 ± 1.5
CK (ukat/l, ULN 2.7)	33.6 ± 58.2	NA
Myoglobin (μg/l, ULN 70)	1345 ± 2422	NA
		
Clinical activity of myositis		
MYOACT global	0.15 ± 0.11	NA
MMT8 (maximum 80)	65.34 ± 12.31	NA
**Autoantibodies (%)**		
Anti-Jo-1	24	NA
Anti-Ro-52	37	NA
Anti-Ro-60	2	NA
Anti-PL-7	5	NA
Anti-PM-Scl-100	12	NA
Anti-PM-Scl-75	7	NA
Anti-Mi-2	12	NA
Anti-Ku	2	NA
Anti-SRP	5	NA
Anti-U1RNP	5	NA
Anti-histones	5	NA
Other (AMA, M2, CENP-B)	2	NA
Negative	21	NA
**Treatment (%)**		
Before therapy	19	0
Treatment less than 1 month	53	0
Treatment more than 1 month	26	0
Glucocorticoids	67	0
Others	19	0

Abbreviations: CK, creatine kinase; CRP, C-reactive protein; F, female; HC, healthy controls; IIM, inflammatory myopathies; M, male; MMT8, manual muscle testing of eight muscle groups; MYOACT, disease activity assessment visual analogue scales; N, number; NA, not analysed

### Increased resistin levels and disease activity in patients with inflammatory myopathies

**Figure 1:**
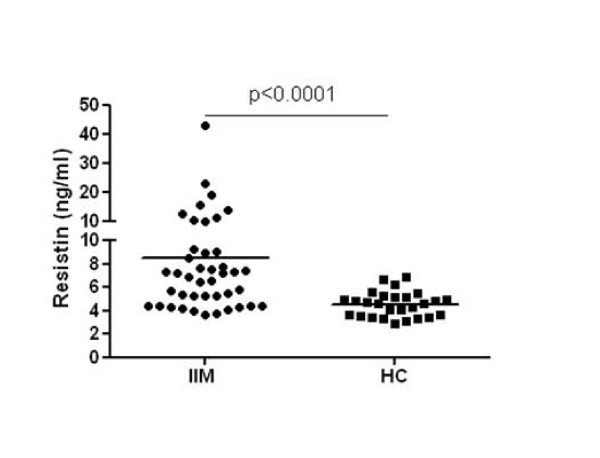
**Increased serum resistin levels in patients with idiopathic inflammatory myopathies (IIM) compared with healthy controls (HC)**.

The serum resistin levels were significantly higher in patients with inflammatory myopathies than in healthy controls (8.53 ± 6.84 ng/ml vs. 4.54 ± 1.08 ng/ml, *P *< 0.0001, Figure 1). Patients with both DM (7.39 ± 3.75 ng/ml, *P *< 0.01) and overlap myositis (9.65 ± 8.88 ng/ml, *P *< 0.001) had significantly higher levels of resistin compared to healthy controls. There were no significant differences in the levels of serum resistin between patients with DM and other types of myositis (7.39 ± 3.75 vs. 9.31 ± 8.30 ng/ml, *P *= 0.626) or between anti-Jo-1 positive and anti-Jo-1 negative patients (9.459 ± 5.948 vs. 8.242 ± 7.153, *P *= 0.281). The serum levels of resistin were comparable between male and female patients (7.160 ± 3.363 vs. 7.864 ± 4.459, *P *= 0.761) and were not affected by age (r = -0.154, *P *= 0.331) or disease duration (r = -0.104, *P *= 0.513). Furthermore, resistin serum levels did not differ significantly between patients treated with glucocorticoids for less and more than one month, and treatment with other immunosuppressive agents also did not affect the serum resistin levels. The resistin levels were not significantly different between patients with and without arthritis or patients with and without interstitial lung disease although these compartments may be a potential source of resistin.

In patients with inflammatory myopathies, but not in healthy controls, the serum resistin levels correlated with the CRP levels (r = 0.328, *P *= 0.040) and, interestingly, positively correlated with the global MYOACT score (r = 0.382, *P *= 0.026). In contrast, resistin levels correlated neither with the creatine kinase and myoglobin levels nor with the MMT8 score in patients with inflammatory myopathies. Furthermore, we found that resistin serum levels significantly correlated with CRP levels (r = 0.717, *P *= 0.037) and MYOACT (r = 0.798, *P *= 0.007) in anti-Jo-1 positive patients, but not in anti-Jo-1 negative patients (CRP r = 0.140, *P *= 0.469; MYOACT r = 0.283, *P *= 0.181). In addition, there was a trend towards correlation between serum resistin levels and myoglobin (r = 0.650, *P *= 0.067) in anti-Jo-1 positive patients.

In patients with overlap myositis, resistin serum levels correlated with CRP (r = 0.511, *P *= 0.030), but not with other measures. In patients with DM, serum resistin levels significantly correlated with MYOACT score (r = 0.667, *P *= 0.001), creatine kinase (r = 0.739, *P *= 0.001) and myoglobin levels (r = 0.791, *P *= 0.0003) and showed a trend towards correlation with CRP levels (r = 0.447, *P *= 0.067). Correlations between the levels of serum resistin and parameters of clinical myositis activity are summarized in Table 2.

**Table 2:** Correlations between serum levels of resistin and disease activity in patients with inflammatory myopathies.

		IIM			Myositis		DM
Parameter	Correlation	all	Jo-1 +	Jo-1-	pure	overlap	
**MYOACT**	**r**	0.382	0.789	0.283	0.866	0.387	0.667
	**p**	0.026	0.007	0.181	0.33	0.125	0.001
							
**MMT8**	**r**	-0.055	-0.179	0.002	-0.400	0.020	-0.439
	**p**	0.765	0.632	0.993	0.750	0.944	0.134
							
**Myoglobin**	**r**	0.233	0.650	0.114	0.200	0.079	0.791
	**p**	0.159	0.067	0.490	0.917	0.794	0.0003
							
**CK**	**r**	0.145	0.417	0.122	0.200	-0.110	0.739
	**p**	0.385	0.270	0.528	0.917	0.663	0.001
							
**CRP**	**r**	0.328	0.717	0.14	-0.800	0.511	0.447
	**p**	0.044	0.037	0.469	0.333	0.030	0.067

Abbreviations: CK, creatine kinase; CRP, C-reactive protein; DM, dermatomyositis; IIM, inflammatory myopathies; Jo-1, anti-Jo-1 antibody; MYOACT, disease activity assessment visual analogue scales

### Expression of resistin in muscle tissue

**Figure 2:**
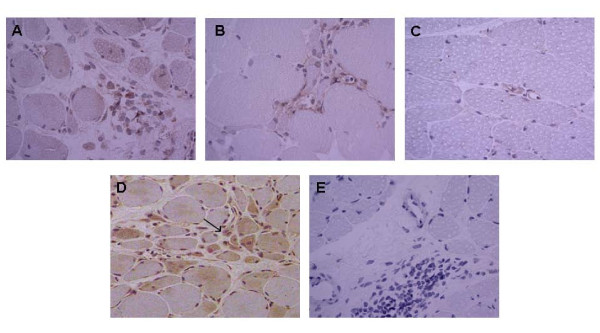
**Expression of resistin in muscle tissue**. Expression of resistin in affected skeletal muscle tissue from patients with dermatomyositis **(a) **and myositis **(b) **in contrast with no expression in the muscle tissue of myasthenia gravis **(c)**. Expression of resistin in regenerating muscle fibres (pointed with arrow) in a patient with dermatomyositis **(d)**. Corresponding tissue sections stained with isotype antibodies **(e)**. Resistin appears as brown. Nuclei were stained with hematoxylin. Original magnification, ×400 (a-c, e) and ×200 (d).

The assessment of resistin staining intensity in inflammatory infiltrates of the sections from muscle biopsies revealed a mean intensity of 1.8 in myositis and 1.6 in DM patients on the 0 to 3 scale. As expected, no lymphocytes were present in control individuals with MG. Therefore, muscle tissue samples from MG patients were used as non-inflammatory controls. We found that resistin expression in muscle tissues from patients with inflammatory myopathies was significantly higher when compared with control muscle tissues from patients with MG (Figure 2). The increased expression of resistin was particularly localised in scattered mononuclear cells and in mononuclear cells in inflammatory infiltrates surrounding large vessels and muscle fibres in patients with inflammatory myopathies. Mild resistin expression was observed in the cytoplasm of some muscle fibres. Resistin staining was observed in regenerating muscle fibres in four-fifths of myositis and three-fifths of DM patients, but not in control patients with MG (Figure 2). Identification of regenerating muscle fibers was based upon hematoxylin and eosin staining. Regenerating muscle fibers were smaller and splitting, with centrally located vesicular myonuclei. The cytoplasm of regenerating muscle fibers appeared basophilic.

### Effects of resistin on mononuclear cells

**Figure 3:**
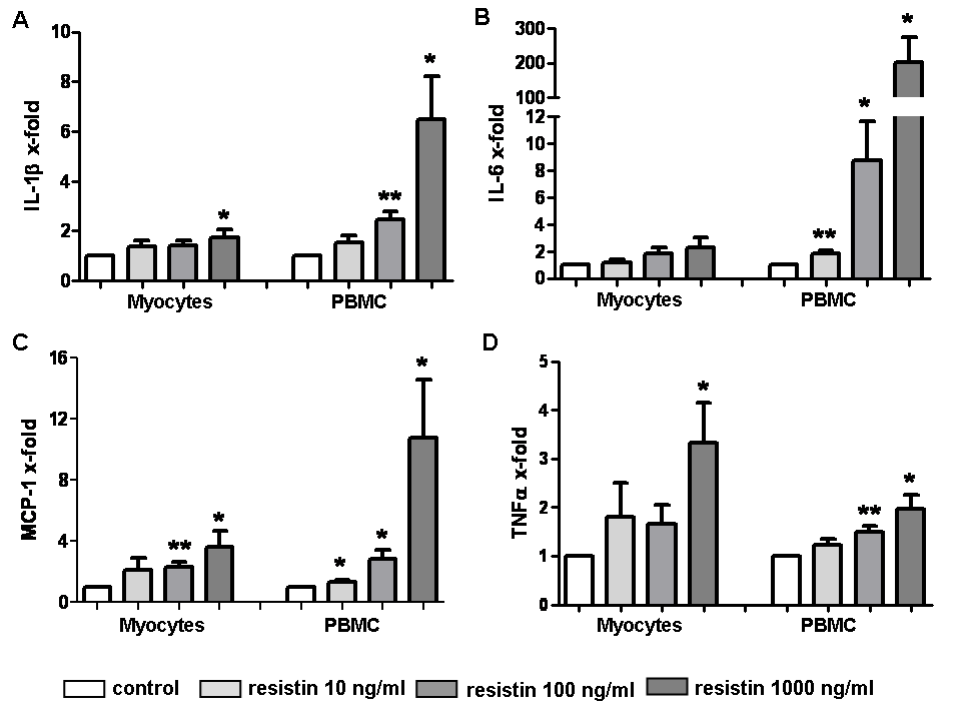
**Effects of resistin on inflammatory response in myocytes and peripheral blood mononuclear cells (PBMC)**. Expression of IL-1α **(a)**, IL-6 **(b)**, MCP-1 **(c) **and TNFα **(d) **mRNA levels after stimulation with human resistin (10, 100, 1,000 ng/ml) for six hours in myocytes and PBMC. Data are shown as fold changes compared to unstimulated controls (in graphs represented by control bar, rated as 1). Bars represent the mean + SEM. *P*-values less than 0.05 were considered statistically significant; * *P *< 0.05, ** *P *< 0.01.

**Figure 4:**
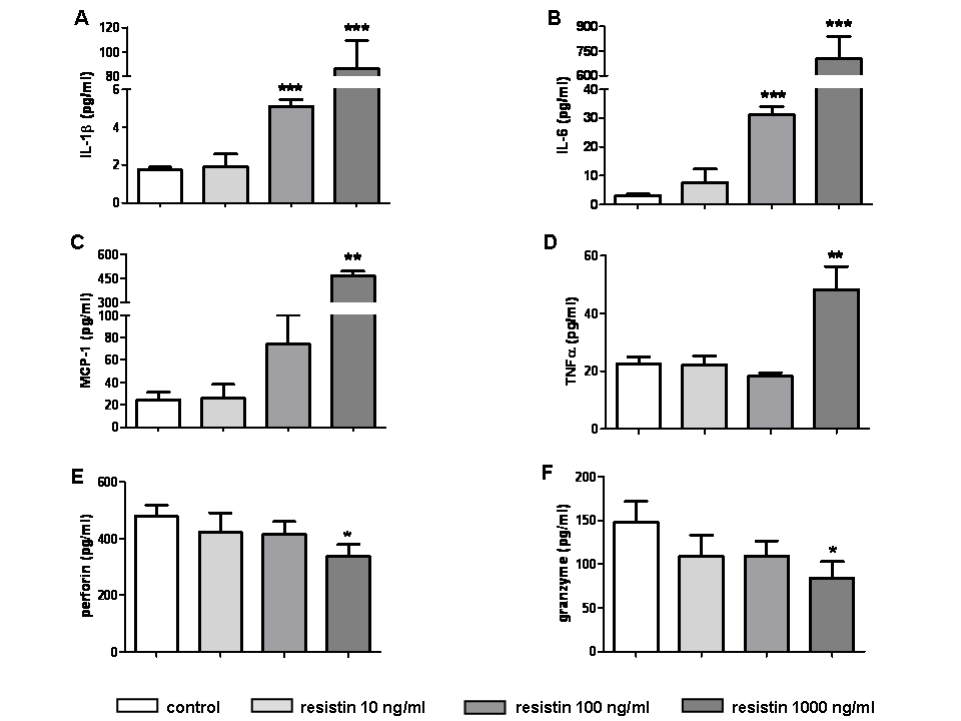
**Effects of resistin on peripheral blood mononuclear cells (PBMC)**. Resistin induces the release of IL-1α **(a)**, IL-6 **(b)**, MCP-1 **(c) **and TNFα **(d) **and reduces the release of perforin **(e) **and granzyme **(f) **from PBMCs into cell culture media after 48 hours. Bars represent the mean + SEM. *P-*values less than 0.05 were considered statistically significant; * *P *< 0.05,** *P *< 0.01, *** *P *< 0.001.

PBMC (*n *= 8) were stimulated with increasing concentrations of resistin (0, 10, 100, 1,000 ng/ml) for 6 or 48 h. As shown in Figure 3, treatment of PBMC with resistin resulted in a significant and dose-dependent induction of IL-1β (up to 6.5-fold), IL-6 (up to 203-fold) and MCP-1 (up to 10.8-fold). The induction of TNFα at the mRNA level was only mild (up to two-fold). This expression pattern was followed by the increased cytokine release into the cell culture supernatants (Figure 4a-d). Cytokine levels after stimulation with the lowest doses of resistin were comparable with those of unstimulated cells but these values were significantly different after stimulation with higher doses of resistin. To study the effect of resistin on the cytotoxic activity of PBMC, the perforin and granzyme levels were analysed in cell culture supernatants. Only the highest concentrations of resistin resulted in a reduction in the release of both perforin and granzyme (Figure 4e, f).

To exclude endotoxin contamination of human recombinant resistin, PBMC (*n *= 3) were stimulated with resistin (1,000 ng/ml) alone or in combination with polymyxin B sulphate (5 μg/ml) for 6 h. There were no differences in the expression of the abovementioned cytokines, indicating that there was no nonspecific effect mediated by recombinant resistin (data not shown).

### Effects of resistin on myocytes

To assess the ability of resistin to induce a possible inflammatory response in skeletal muscle cells, as it does in mononuclear cells, myocytes (*n *= 9) were stimulated with increasing concentrations of resistin (0, 10, 100, 1,000 ng/ml). Although the treatment of myocytes with resistin for 6 hours contributed to the slightly increased gene expression of some pro-inflammatory cytokines (Figure 3), the release of these cytokines into the cell culture supernatants after 48 hours from stimulated and unstimulated cells did not significantly differ. There was also no resistin-associated effect of on the expression of several type I interferon induced genes, such as CXCL10, IFI27, IFI44, IFI44L, RSAD2, OAS1, ISG15, IFIT1, MX1 (data not shown). Furthermore, as assessed by MTT assay, resistin did not affect proliferation of skeletal muscle cells *in vitro*.

## Discussion

In this study, we report for the first time an association between increased levels of serum resistin and the disease activity of patients with inflammatory myopathies, particularly in anti-Jo-1 positive and dermatomyositis patients. Additionally, we found that the expression of resistin is up-regulated in muscle tissues of patients with inflammatory myopathies. Lastly, we found that resistin may contribute to the increased production of pro-inflammatory cytokines in mononuclear infiltrates, thus indirectly participating in muscle tissue pathology.

Although resistin was initially associated with metabolic disorders, increased levels of resistin and its positive correlation with inflammatory markers and disease activity have been previously demonstrated in patients with rheumatoid arthritis (RA) [4,18-20]. In a study by Almehed and colleagues, serum resistin levels did not differ between patients with systemic lupus erythematosus (SLE) and healthy controls [21]; whereas in another study, SLE was independently associated with higher resistin levels [22]. Moreover, in both studies, the levels of resistin positively correlated with inflammatory markers, disease-specific measures and renal dysfunction. In our study, we observed a strong correlation between higher levels of serum resistin and CRP and, most importantly, we observed an association with the global disease activity assessment of inflammatory myopathy. Importantly, we found that serum resistin levels were strongly associated with CRP and global disease activity, and a trend was also observed towards correlation between resistin levels and myoglobin in patients with myositis-specific anti-Jo-1 antibody in contrast to anti-Jo-1 negative patients. Furthermore, resistin levels significantly correlated with global disease activity and muscle enzymes in DM patients. These results are in line with the abovementioned findings, further supporting an association between resistin and autoimmune rheumatic diseases [4,18,22]. On the other hand, serum resistin levels did not correlate with muscle weakness. Thus, it could be hypothesized that serum resistin concentration may reflect global disease activity, including extramuscular organ involvement, rather than functional impairment in inflammatory myopathies. However, disease specific mechanisms can be suggested.

Recently, we demonstrated the increased expression of resistin in immune cells of RA synovial tissue [19]. In the present study, we consistently observed the up-regulation of resistin in the mononuclear cells of inflammatory infiltrates surrounding vessels and muscle fibres in patients with inflammatory myopathies. In contrast, there was no expression of resistin in control non-inflammatory muscle tissue. Importantly, a majority of samples from DM and myositis patients showed resistin staining in regenerating muscle fibres, perhaps implicating a contribution of the muscle tissue to the inflammatory process or influence of resistin on regenerating/immature muscle precursors in the pathogenesis of myositis. The presence of fibre degeneration and regeneration is a typical histopathological picture of inflammatory myositis. Initially, muscle repair is characterized by inflammation and degeneration of damaged muscle tissue. It is followed by activation, proliferation and differentiation of myogenic, so-called satellite cells with subsequent fusion and formation of multinucleated muscle fibres. This process requires a crosstalk between immune and muscle cells, including secreted factors [23,24]. In fact, at the moment it is not possible to distinguish the features of the inflammatory myositis that promote the injury from those that cause muscle regeneration and repair [25]. Accordingly, the role of resistin in the pathology of muscle tissue in myositis is underpinned by its association with parameters of disease activity in the group of patients with myositis associated autoantibody Jo-1. As we found increased systemic and local levels of resistin in inflammatory myopathies, we examined the effect of resistin on muscle and mononuclear cells *in vitro*. In line with previous reports [8,26], we found that resistin induces expression and synthesis of several proinflammatory mediators in mononuclear cells, thus possibly contributing to muscle tissue pathology. As demonstrated previously, IL-1, IL-6, MCP-1, as well as TNFα, are increased in myositis muscle tissues and contribute to disease pathogenesis [27-29]. However, these cytokines may have dual function and may not only contribute to muscle tissue damage, but also to the regeneration and healing of the muscle tissue [25,30]. Considering the ambivalent role of several mediators in inflammatory myositis, dual functions of resistin may be suggested. In this regard, we observed a trend towards the reduced synthesis of the cytotoxic enzymes perforin and granzyme in resistin-stimulated immune cells.

It has been shown that several Toll-like receptors (TLRs) are expressed in immune cells but are not present in the muscle fibres of patients with inflammatory myopathies [31]. A recent study demonstrated that a TLR4 receptor mediates the proinflammatory effects of resistin in human cells [32]. Therefore, we assume that the lack of resistin-associated effects on myocytes in our study may be due to the absence of TLR4 receptors on the surface of these cells. That being said, resistin did not modulate the expression of several interferon (IFN)-α/β induced genes that have been recently observed in tissue of patients with inflammatory myopathies [33]. Interestingly, immature muscle precursors in myositis biopsy tissues have been recently demonstrated as an important source of IFN-β, which was, however, mediated by TLR-3 activation [34].

## Conclusion

We have demonstrated that increased levels in tissue as well as serum concentration of resistin in patients with inflammatory myopathies correlate with global disease activity. In patients with myositis-specific anti-Jo-1 antibody and, particularly, in dermatomyositis patients, elevated resistin levels associated with disease activity and muscle enzymes. We suggest that resistin indirectly participates in muscle tissue damage by inducing the production of pro-inflammatory cytokines by mononuclear cells. The exact role of resistin in muscle tissue regeneration or destruction in inflammatory myopathies needs further study.

## Abbreviations

ADSF: adipocyte-secreted factor; CRP: C-reactive protein; DM: dermatomyositis; F: female; FIZZ3: inflammatory zone 3; HC: healthy controls; IFN: interferon; IIM: inflammatory myopathies; IL: interleukin; M: male; MCP-1: monocyte chemoattractant protein 1; MG: myasthenia gravis; MMT8: manual muscle testing of eight muscle groups; MRI: magnetic resonance imaging; MYOACT: myositis disease activity assessment visual analogue scales; NA: not analysed; PBMC: peripheral blood mononuclear cells; PM: polymyositis; RA: rheumatoid arthritis; SLE: systemic lupus erythematosus; TBS: tris buffered saline; TLR: Toll-like receptors; TNF: tumour necrosis factor.

## Competing interests

The authors declare that they have no competing interests.

## Authors' contributions

MF performed the majority of the *in vitro *experiments and the statistical analysis, in addition to preparing the manuscript. HH performed the laboratory measurements. KK performed the immunohistochemistry and was involved in enrolling the patients. LAC and MK contributed to *in vitro *experiments. LP, HM, JZ and JV were involved in enrolling the patients and their clinical data. SG and JV assisted in the design of the study. LS was in charge of the design and conception of the study, helped with the interpretation of the data and with drafting the manuscript. All authors read and approved the final manuscript.

## References

[B1] Dalakas MC Hohlfeld R Polymyositis and dermatomyositis Lancet 2003 362 971 982 10.1016/S0140-6736(03)14368-1 14511932

[B2] De Paepe B Creus KK De Bleecker JL Role of cytokines and chemokines in idiopathic inflammatory myopathies Curr Opin Rheumatol 2009 21 610 616 10.1097/BOR.0b013e3283317b31 19726994

[B3] Filková M Haluzík M Gay S Senolt L The role of resistin as a regulator of inflammation: Implications for various human pathologies Clin Immunol 2009 133 157 170 10.1016/j.clim.2009.07.013 19740705

[B4] Bokarewa M Nagaev I Dahlberg L Smith U Tarkowski A Resistin, an adipokine with potent proinflammatory properties J Immunol 2005 174 5789 5795 15843582 10.4049/jimmunol.174.9.5789

[B5] Anderson PD Mehta NN Wolfe ML Hinkle CC Pruscino L Comiskey LL Tabita-Martinez J Sellers KF Rickels MR Ahima RS Reilly MP Innate immunity modulates adipokines in humans J Clin Endocrinol Metab 2007 92 2272 2279 10.1210/jc.2006-2545 17374708

[B6] Lehrke M Reilly MP Millington SC Iqbal N Rader DJ Lazar MA An inflammatory cascade leading to hyperresistinemia in humans PLoS Med 2004 1 e45 10.1371/journal.pmed.0010045 15578112 PMC529430

[B7] Kaser S Kaser A Sandhofer A Ebenbichler CF Tilg H Patsch JR Resistin messenger-RNA expression is increased by proinflammatory cytokines *in vitro* Biochem Biophys Res Commun 2003 309 286 290 10.1016/j.bbrc.2003.07.003 12951047

[B8] Nagaev I Bokarewa M Tarkowski A Smith U Human resistin is a systemic immune-derived proinflammatory cytokine targeting both leukocytes and adipocytes PLoS ONE 2006 1 e31 10.1371/journal.pone.0000031 17183659 PMC1762367

[B9] Mu H Ohashi R Yan S Chai H Yang H Lin P Yao Q Chen C Adipokine resistin promotes *in vitro *angiogenesis of human endothelial cells Cardiovasc Res 2006 70 146 157 10.1016/j.cardiores.2006.01.015 16515776

[B10] Verma S Li SH Wang CH Fedak PW Li RK Weisel RD Mickle DA Resistin promotes endothelial cell activation: further evidence of adipokine-endothelial interaction Circulation 2003 108 736 740 10.1161/01.CIR.0000084503.91330.49 12874180

[B11] Kawanami D Maemura K Takeda N Harada T Nojiri T Imai Y Manabe I Utsunomiya K Nagai R Direct reciprocal effects of resistin and adiponectin on vascular endothelial cells: a new insight into adipocytokine-endothelial cell interactions Biochem Biophys Res Commun 2004 314 415 419 10.1016/j.bbrc.2003.12.104 14733921

[B12] Tomasová Studynková J Charvát F Jarosová K Vencovsky J The role of MRI in the assessment of polymyositis and dermatomyositis Rheumatology (Oxford) 2007 46 1174 1179 10.1093/rheumatology/kem088 17500079

[B13] Dalakas MC Polymyositis, dermatomyositis and inclusion-body myositis N Engl J Med 1991 325 1487 1498 1658649 10.1056/NEJM199111213252107

[B14] Amato AA Griggs RC Unicorns, dragons, polymyositis, and other mythological beasts Neurology 2003 61 288 289 10.1212/WNL.61.3.288 12913184

[B15] Troyanov Y Targoff IN Tremblay JL Goulet JR Raymond Y Senécal JL Novel classification of idiopathic inflammatory myopathies based on overlap syndrome features and autoantibodies: analysis of 100 French Canadian patients Medicine (Baltimore) 2005 84 231 249 10.1097/01.md.0000173991.74008.b0 16010208

[B16] Isenberg DA Allen E Farewell V Ehrenstein MR Hanna MG Lundberg IE Oddis C Pilkington C Plotz P Scott D Vencovsky J Cooper R Rider L Miller F International Myositis and Clinical Studies Group (IMACS) International Myositis and Clinical Studies Group (IMACS). International consensus outcome measures for patients with idiopathic infl ammatory myopathies. Development and initial validation of myositis activity and damage indices in patients with adult onset disease Rheumatology(Oxford) 2004 43 49 54 10.1093/rheumatology/keg427 12867580

[B17] Rider LG Koziol D Giannini EH Jain MS Smith MR Whitney-Mahoney K Feldman BM Wright SJ Lindsley CB Pachman LM Villalba ML Lovell DJ Bowyer SL Plotz PH Miller FW Hicks JE Validation of manual muscle testing and a subset of eight muscles for adult and juvenile idiopathic inflammatory myopathies Arthritis Care Res (Hoboken) 2010 62 465 472 10.1002/acr.20035 20391500 PMC2924143

[B18] Schäffler A Ehling A Neumann E Herfarth H Tarner I Schölmerich J Müller-Ladner U Gay S Adipocytokines in synovial fluid JAMA 2003 290 1709 1710 10.1001/jama.290.13.1709-c 14519703

[B19] Senolt L Housa D Vernerová Z Jirásek T Svobodová R Veigl D Anderlová K Müller-Ladner U Pavelka K Haluzík M Resistin in rheumatoid arthritis synovial tissue, synovial fluid and serum Ann Rheum Dis 2007 66 458 463 17040961 10.1136/ard.2006.054734 PMC1856051

[B20] Migita K Maeda Y Miyashita T Kimura H Nakamura M Ishibashi H Eguchi K The serum evels of resistin in rheumatoid arthritis patients Clin Exp Rheumatol 2006 24 698 701 17207388

[B21] Almehed K d'Elia HF Bokarewa M Carlsten H Role of resistin as a marker of inflammation in systemic lupus erythematosus Arthritis Res Ther 2008 10 R15 10.1186/ar2366 18234104 PMC2374439

[B22] Baker JF Morales M Qatanani M Cucchiara A Nackos E Lazar MA Teff K Von Feldt JM Resistin levels in lupus and associations with disease-specific measures, insulin resistance, and coronary calcification J Rheumatol 2011 38 2369 2375 10.3899/jrheum.110237 21885493 PMC5702914

[B23] Karalaki M Fili S Philippou A Koutsilieris M Muscle regeneration: cellular and molecular events In Vivo 2009 23 779 796 19779115

[B24] Tidball JG Villalta SA Regulatory interactions between muscle and the immune system during muscle regeneration Am J Physiol Regul Integr Comp Physiol 2010 298 R1173 1187 10.1152/ajpregu.00735.2009 20219869 PMC2867520

[B25] Loell I Lundberg IE Can muscle regeneration fail in chronic inflammation: a weakness in inflammatory myopathies? J Intern Med 2011 269 243 257 10.1111/j.1365-2796.2010.02334.x 21205023

[B26] Bokarewa M Nagaev I Dahlberg L Smith U Tarkowski A Resistin, an adipokine with potent proinflammatory properties J Immunol 2005 174 5789 5795 15843582 10.4049/jimmunol.174.9.5789

[B27] Liprandi A Bartoli C Figarella-Branger D Pellissier JF Lepidi H Local expression of monocyte chemoattractant protein-1 (MCP-1) in idiopathic inflammatory myopathies Acta Neuropathol 1999 97 642 648 10.1007/s004010051041 10378384

[B28] Lundberg I Kratz AK Alexanderson H Patarroyo M Decreased expression of interleukin-1alpha, interleukin-1beta, and cell adhesion molecules in muscle tissue following corticoid treatment in patients with polymyositis and dermatomyositis Arthritis Rheum 2000 43 336 348 10.1002/1529-0131(200002)43:2<336::AID-ANR13>3.0.CO;2-V 10693873

[B29] Bilgic H Ytterberg SR Amin S McNallan KT Wilson JC Koeuth T Ellingson S Newman B Bauer JW Peterson EJ Baechler EC Reed AM Interleukin-6 and type I interferon-regulated genes and chemokines mark disease activity in dermatomyositis Arthritis Rheum 2009 60 3436 3446 10.1002/art.24936 19877033

[B30] Mackiewicz Z Hukkanen M Povilenaite D Sukura A Fonseca JE Virtanen I Konttinen YT Dual effects of caspase-1, interleukin-1 beta, tumour necrosis factor-alpha and nerve growth factor receptor in inflammatory myopathies Clin Exp Rheumatol 2003 21 41 48 12673888

[B31] Kim GT Cho ML Park YE Yoo WH Kim JH Oh HJ Kim DS Baek SH Lee SH Lee JH Kim HY Kim SI Expression of TLR2, TLR4, and TLR9 in dermatomyositis and polymyositis Clin Rheumatol 2010 29 273 279 10.1007/s10067-009-1316-7 19953283 PMC2812423

[B32] Tarkowski A Bjersing J Shestakov A Bokarewa MI Resistin competes with lipopolysaccharide for binding to toll-like receptor 4 J Cell Mol Med 2010 14 1419 1431 19754671 10.1111/j.1582-4934.2009.00899.x PMC3829009

[B33] Greenberg SA Dermatomyositis and type 1 interferons Curr Rheumatol Rep 2010 12 198 203 10.1007/s11926-010-0101-6 20425524 PMC2929916

[B34] Tournadre A Lenief V Eljaafari A Miossec P Immature muscle precursors are a source of interferon-β in myositis: role of Toll-like receptor 3 activation and contribution to HLA class I up-regulation Arthritis Rheum 2012 64 533 541 22094963 10.1002/art.33350

